# Neurotensin pathway in digestive cancers and clinical applications: an overview

**DOI:** 10.1038/s41419-020-03245-8

**Published:** 2020-12-02

**Authors:** Niki Christou, Sabrina Blondy, Valentin David, Mireille Verdier, Fabrice Lalloué, Marie-Odile Jauberteau, Muriel Mathonnet, Aurélie Perraud

**Affiliations:** 1 Laboratoire EA3842 CAPTuR « Contrôle de l’Activation cellulaire, Progression Tumorale et Résistances thérapeutiques », Faculté de médecine, 2 rue du Docteur Marcland, 87025 Limoges, France; 2 Service de Chirurgie Digestive, Endocrinienne et Générale, CHU de Limoges, 2 avenue Martin Luther King, 87042 Limoges, France; 3 Service de Pharmacie, CHU de Limoges, 2 avenue Martin Luther King, 87042 Limoges, France; 4 Service d’Immunologie, CHU de Limoges, 2 avenue Martin Luther King, 87042 Limoges, France

**Keywords:** Cancer, Gastrointestinal cancer

## Abstract

Initially, NEUROTENSIN (NTS) has been shown to play physiological and biological functions as a neuro-transmitter/modulator in the central nervous system and as an endocrine factor in the periphery, through its binding to two kinds of receptors: NTSR1 and 2 (G protein-coupled receptors) and NTSR3/sortilin (a vacuolar protein-sorting 10-domain receptor). NTS also plays oncogenic roles in many types of cancer, including digestive cancers. In tumor tissues, NTS and NTSR1 expression is higher than in healthy ones and is associated with poor prognosis. NTS and NTRS1 promote cancer progression and play key functions in metastatic processes; they modulate several signaling pathways and they contribute to changes in the tumor microenvironment. Conversely, NTRS2 involvement in digestive cancers is poorly understood. Discovered for mediating NTS biological effects, sortilin recently emerged as a promising target as its expression was found to be increased in various types of cancers. Because it can be secreted, a soluble form of sortilin (sSortilin) appears as a new serum biomarker which, on the basis of recent studies, promises to be useful in both the diagnosis and tumor progression monitoring. More precisely, it appears that soluble sortilin can be associated with other receptors like TRKB. These associations occur in exosomes and trigger the aggressiveness of cancers like glioblastoma, leading to the concept of a possible composite theranostic biomarker. This review summarizes the oncogenic roles of the NTS signaling pathways in digestive cancers and discusses their emergence as promising early diagnostic and/or prognostic biomarkers and therapeutic targets.

## Facts

- Digestive cancers are frequent; colorectal cancer is the third most common cancer worldwide.
- NEUROTENSIN (NTS) known as both a neurotransmitter/modulator in the central nervous system and a hormone in the periphery, has recently been shown to be involved in the digestive carcinogenesis process.
- NTS and its receptors, NTSR1–2 and sortilin/NTSR3 might emerge as potential diagnostic, prognostic, and therapeutic biomarkers.

## Open questions

- What NTS and their receptors correspond to?
- What are their roles in physiological processes?
- What are their roles in pathological processes such as cancers and, specifically, digestive cancers?

## Introduction

**Fig. 1:**
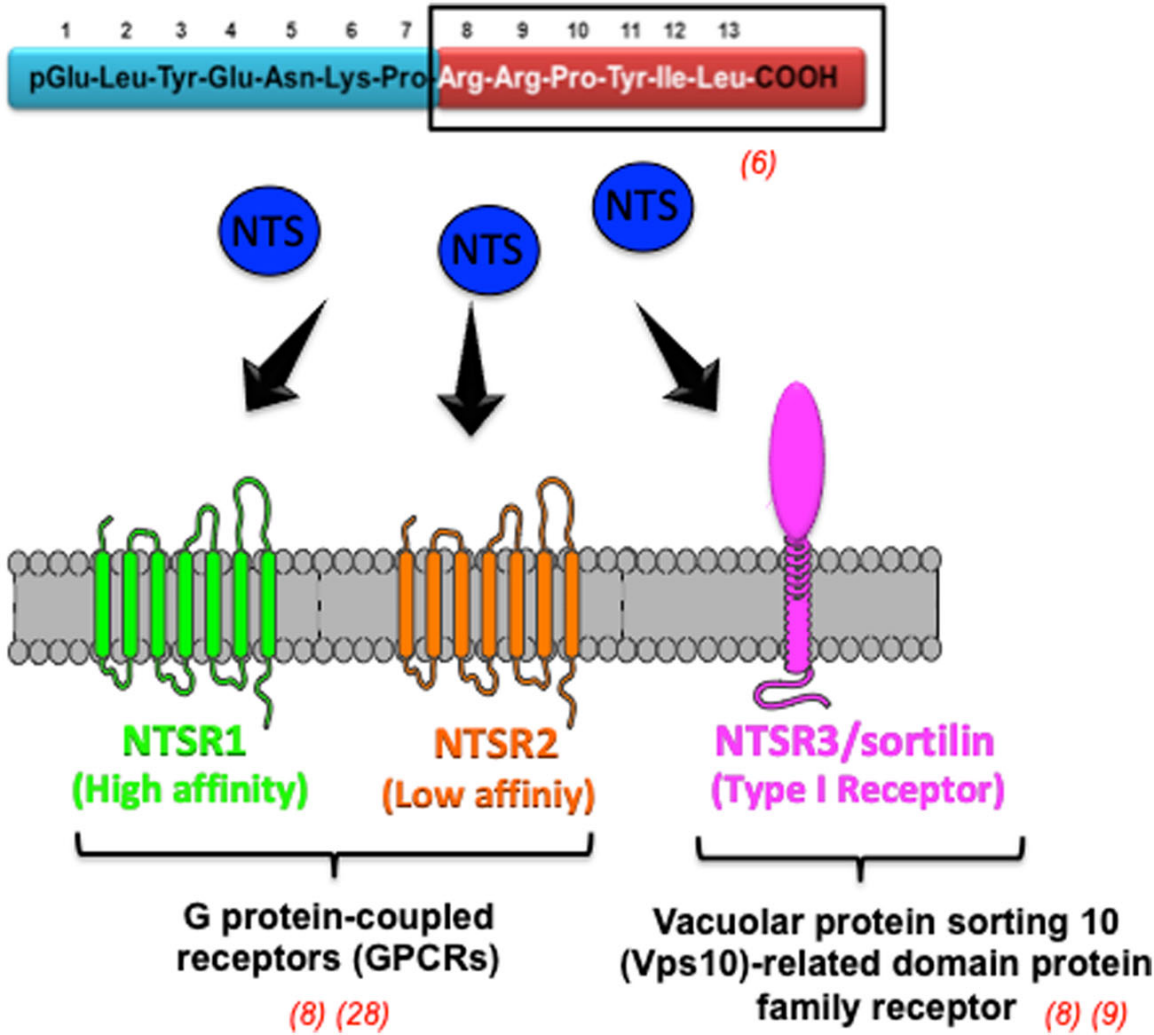
Receptors of neurotensin (NTSRs). Three receptors of NTS are known: two of them are G-protein-coupled receptors (GPCRs): NTSR1 with high affinity for NTS, and NTSR2 with low affinity. The third one is a Vacuolar protein sorting 10 (Vps10) related domain protein family receptor named NTSR3 (sortilin).

NEUROTENSIN (NTS) is a neurotransmitter/modulator in the central nervous system and a hormone in the periphery^1,2^. It is released by endocrine cells in the intestinal mucosa, metabolized by the liver and is involved in several functions of the gastrointestinal tract^3^. NTS is able to inhibit gastric acid secretion and to modulate gut motility, depending on the type of muscle and its localization (ascending/descending colon, circular, and other smooth muscles) but it also acts on mucosa proliferation^4,5^. Cell growth in digestive organs (stomach, colon, pancreas, and small bowel mucosa) is possible thanks to NTS^6^ stimulation through its binding to two G protein-coupled receptors (GPCRs), neurotensin receptors (NTSR) 1 and 2, and through a third receptor, NTSR3/sortilin^7–9^, a member of the vacuolar protein sorting 10 (Vps10)-related domain-containing protein family^10^ (Fig. 1). Vps10 domain-containing receptors, which are typical transmembrane proteins, include three members with sortilin, a sorting-related receptor with A-type repeats (sorLA) and sortilin-related Vps10 domain-containing receptors. Sortilin, the most studied of them, is known to be involved in neurodegenerative and inflammatory diseases and immunologic processes as well, through its key role in protein cell sorting, trafficking, and releasing, including NTS and NTSR1^11,12^. A soluble form of sortilin (sSortilin)^13^ has also been detected and identified as a promising biomarker in CRC^14,15^. Initially reported in breast carcinoma^16,17^, NTS and its receptors are also linked to the development and progression of multiple cancer types, including digestive cancers^18^. Recently, a clinical trial has highlighted increased NTS plasma levels in patients with colon cancer in comparison with healthy individuals and especially those bearing noncancerous polyps, thus suggesting that these levels might be of diagnosis value^19^. In addition, NTS pathway emerged to play crucial roles in migration and invasion processes, which are prerequisites to metastatic spread, and in inflammation processes associated with tumor microenvironment modulation^20^. Hence, they could constitute potential targets for the development of anticancer drugs^21–23^. A recent patent (US20170218057A1) has been deposited that relates an antineurotensin long fragment antibody, which is able to bind NTS with high affinity and to neutralize its oncogenic properties^24^. However, this antibody has not yet been tested on colorectal cancer cells.

This review focuses on NTS and its receptors, NTSR1–2 and sortilin/NTSR3. It aims to underline their potential use as diagnostic, prognostic and therapeutic biomarkers and targets in digestive (gastric, pancreatic, hepatic, and colorectal) cancers.

## NTS and NTSRs: related signaling pathways and biological processes

NTS is a 13 amino acids (aa) neuropeptide evidenced in 1973 by Carraway and Leeman in bovine hypothalamus extracts and identified in neurodegenerative diseases (Parkinson and Huntington diseases)^1^. More recently, it has also been detected in the neuroendocrine system of the gastro-intestinal tract (oesophageal gastric, pancreatic, and colonic cells), where it exerts secretory^25^, proliferative/trophic and motility actions^4^. NTS biological effects are mainly mediated by two GPCRs^26^: NTSR1 (410 aa, 56 kDa), the high-affinity NTS receptor (Kd = 0.15–0.5 nM), and NTSR2 (418 aa, 62 kDa), the low-affinity NTS receptor (Kd = 5–7 nM), which possesses 64% homology with NTSR1.

**Fig. 2:**
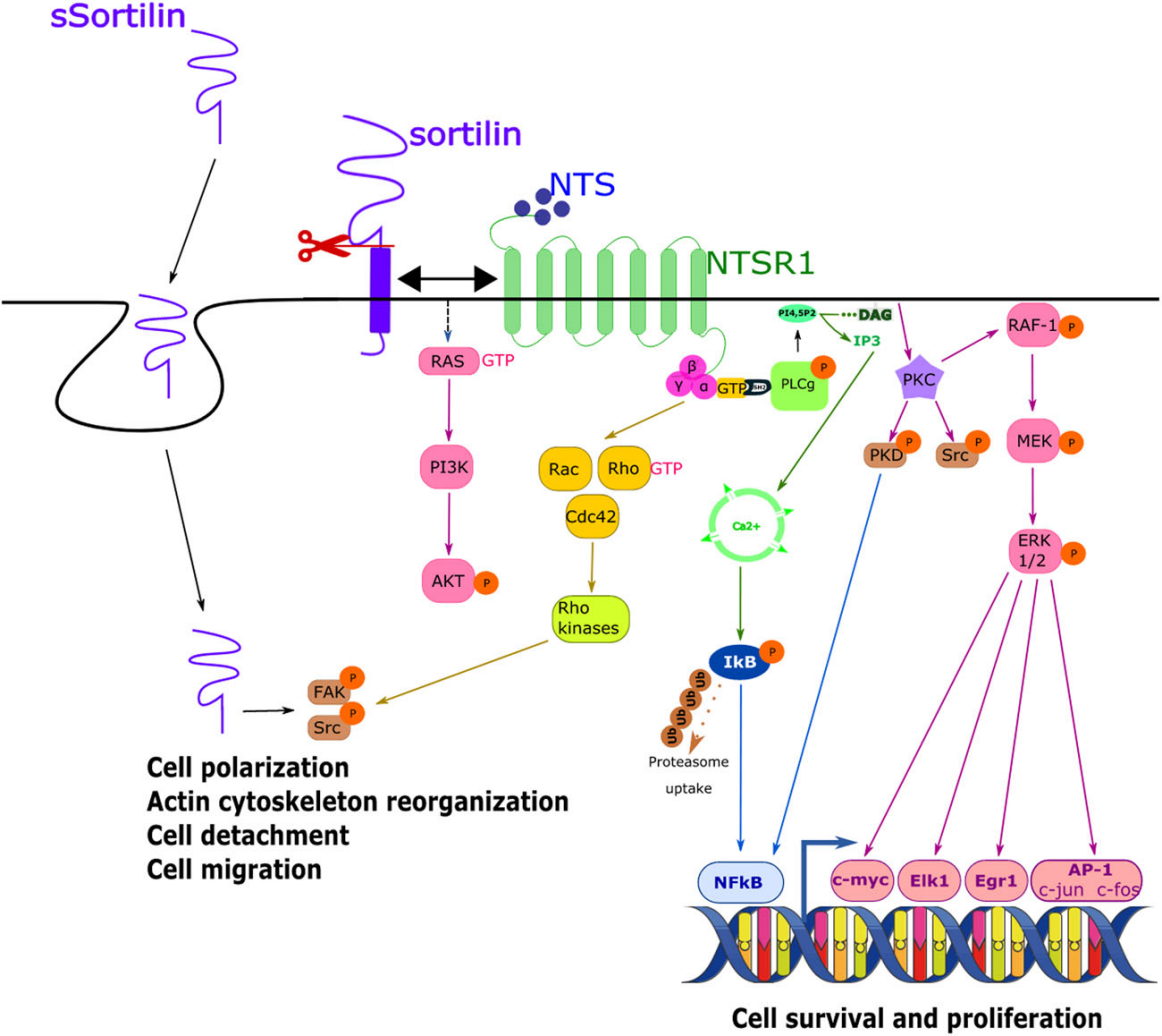
Simplified scheme of the NTS signaling pathways involving NTSR1 and sortilin, and biological effects associated. NTS binds to NTSR1, either alone or complexed with sortilin at the cell membrane, and activates four main pathways: (i) Rho GTPases, (ii) intracellular Ca^2+^ release, (iii) PKC/RAF-1/MAPK-ERK1/2, and (iv) PI3K/AKT. Then, each one of these targets activates downstream effectors and transcription factors which participate in the transcription of genes involved in cell survival and proliferation, as well as in cytoskeleton dynamics and cell migration. Sortilin can be cleaved and released from the plasma membrane as a soluble form (sSortilin) that, after being internalized, directly stimulates FAK ERK1/2 extracellular signal–regulated kinases, FAK focal adhesion kinase, MAPK mitogen-activated protein kinase, NTS neurotensin, NTSR1 neurotensin receptor 1, PKC protein kinase C, PI3K phosphtidylinositol 3-kinase, sSortilin soluble form of sortilin.

Whereas NTS/NTSR2 signaling pathway is not yet fully understood, that of NTS/NTSR1 is well known. NTS binding to the Gαq11 subunit of NTSR1 triggers phospholipase C-γ (PLC-γ) activation, whose activity leads to the production of inositol trisphosphate (IP3) and diacylglycerol (DAG) from phosphatidylinositol-4,5-bisphosphate. IP3 and DAG respectively trigger the mobilization of intracellular Ca^2+^ and activation of protein kinase-C (PKC). PKC then activates the mitogen-activated protein kinase (MAPK)-extracellular signal–regulated kinases (ERK1/2) pathway independently of RAS, via direct stimulation of RAF-1. Active PKC also activates protein kinase D which in turn leads to the activation of the nuclear factor-kappa B (NFκB) transcription factor. MAPK-ERK1/2 pathway activates transcription factors, such as the activator protein 1 (AP-1) complex, NFκB, early growth response protein 1 (Egr-1), the ternary complex factor (TCF) Elk-1, and c-myc, allowing cell survival and proliferation^27–29^. NTS also induces activation of Rho GTPases, the focal adhesion kinase (FAK) and the proto-oncogene tyrosine-protein kinase Src, hence modulating cytoskeleton dynamics and cell migration^18^ (Fig. 2).

While most of NTS mitogenic effects are mainly mediated by NTSR1, the roles played by sortilin/NTSR3 in this context are yet unclear. Sortilin (831 aa, 95–110 kDa) is, together with sorLA and sorCS1–3, a member of the Vps10 domain receptor family^21,30^. If sortilin binds a wide variety of ligands including neurotrophins (NT), progranulin, lipoproteins, and apolipoproteins or the glucose transporter isoform 4, it presents a much higher affinity for NTS, which therefore impairs the binding of all of the other ligands^31^. It has been suggested that sortilin is able to directly induce signaling after NTS binding, promoting migration of microglial cells^31^. However, mechanisms by which NTS/sortilin binding induces signal transduction are still unclear and poorly understood. To date, sortilin involvement in the modulation of NTS pro-survival signaling pathway has been mainly evidenced through its three main roles: (i) a scavenger, when expressed to the cell membrane, through the binding, internalization^32^ and degradation of its ligands^30^, (ii) a regulator of intracellular traffic and secretion of its ligands and their receptors^30,33^, (iii) receptor and coreceptor, displacing ligands (including NTS and NT) from their specific receptors^34^, and thus enhancing the associated biological responses^30,35,36^. Like all Vps10-related domain protein family members, sortilin can be cleaved and released as a soluble form (sSortilin) from the plasma membrane through PKC-dependent mechanisms. The roles and functions of sSortilin are not yet completely elucidated. This protein could act as a decoy that would prevent the binding of ligands to their specific receptors, but could also be internalized and/or bound to a yet unknown receptor to participate in the metastatic process in CRC, through FAK activation (Fig. 2), thus activating the phosphoinositide 3-kinase (PI3K)/AKT pathway^15,18,30,37,38^.

Many studies have demonstrated NTS oncogenic activities (cell survival, proliferation, migration, and invasion), in an endocrine, autocrine and/or paracrine manner, in several types of cancers and at each step of cancer progression. NTSR1 is involved in tumor progression in many cancers, including digestive cancers^21,39–41^. NTSR2 has been reported to be involved in prostatic cancer, chronic lymphocytic leukaemia^42,43^ and glioma^44^, but little is known about NTS/NTSR2 action mechanisms involved in the progression of cancers. Finally, alteration of sortilin expression and/or its cellular functions leads to a deregulation of biological processes, thus contributing to the development of lipid disorders, neurodegenerative and inflammatory diseases^12,45–47^ and cancerous diseases^33,48^. Although the NTS pathway has not yet been studied in oesophageal squamous cell carcinoma, numerous studies have been performed on gastric, pancreatic, hepatic and colon tumors, relating (i) how NTS and its receptors could be involved in cancer development and progression, and (ii) why they might be promising pharmacological targets and biomarkers in cancer therapy, as summarized below.

## Oncogenic roles of NTS and NTSR in digestive cancers

### Gastric cancer

Gastric cancer (GC) is the fourth most common malignant tumor in the world^49^ with half of the incidence linked to Asian people. The most frequent type of gastric cancer is adenocarcinoma. Its development is triggered by different factors, especially chronic inflammation with possible infectious substratum (*Helicobacter pylori* infections). Surgical resection, associated with standardized lymphadenectomy, remains the gold standard treatment^50^. While its incidence declines because of higher standards of hygiene, healthier diet and *H. pylori* eradication, this cancer becomes symptomatic only at an advanced stage. Evidencing of new serum biomarkers allowing early diagnosis would be of significant benefit. In addition, the overall survival rate of patients with advanced gastric adenocarcinomas remains low, despite the use of new targeted agents like the human epidermal growth-factor receptor 2 (HER2) antibody (trastuzumab)^51^. It is noteworthy that positive HER2 expression has been detected in less than 20% of these patients^52^. While neither NTSR2 nor sortilin have been studied in GC cells, NTS and NTSR1 expressions are associated with poor prognosis. NTSR1 expression is higher in tumor than in healthy tissues^53,54^, and is correlated with the most advanced pathological grade and pTNM (pathology Tumor Nodes Metastases) stages^55^. However, this latter clinical study by Zygulska et al., does not show any correlation between NTSR1 tumoral tissue expression level and age, sex or Lauren Classification (histological classification of adenocarcinoma between “intestinal-type” and “diffuse-type”). In a previous study, higher NTSR1 expression with higher pTNM grade, corresponding to a diffuse type, according to Lauren’s Classification, associated with a higher pathological grade has been significantly correlated with poorer prognosis. In vitro and in vivo studies have shown NTS and NTSR implication in cancer cell migration and invasion via activation of PKC, ERK1/2 and PI3K signaling pathways. Moreover, NTS plasma concentration and metalloproteinase (MMP)-9 activity, are positively correlated, and found at higher levels in GC patients compared to controls^56^. Inhibition of NTSR1 expression by a NTSR1 antagonist or the knockdown of the NTSR1 gene leads to a significant decrease of MMP-9 expression and activity^54^. These findings suggest that NTSR1 could be a potential therapeutic target for the treatment of gastric cancer.

### Pancreatic cancer

Pancreatic ductal adenocarcinoma (PDAC) is the fourth cause of cancer-related death and is the most lethal of common malignancies in the world with poor 5-year survival rate (less than 10%), due to complex and delayed diagnosis^57^. Thus, therapeutic options are limited, and surgery is not always possible. Better knowledge of risk factors and actors of pancreatic carcinogenesis is needed to get earlier diagnosis and to improve the efficiency of the therapeutic protocols. By 2030, PDAC could become the second leading cause of cancer-related death in the United States^58^.

As early as 1998 it was shown that both NTS and NTSR1 were higher expressed in PDAC tissues as compared with non-cancerous ones^59^, and higher expression levels strongly correlate with increasing pathological grades^60,61^. Moreover, higher NTSR1 expression is found in primary tumors and liver metastases of invasive pancreatic cancers^62^. In PDAC cell lines, NTS stimulates ERK1/2 and AKT pathways dependently of PKC activation and independently of EGFR transactivation^63–65^. NTS-mediated PKC activation stimulates RAF-1, which activates the MAPK-ERK1/2 cascade; phosphorylated ERK1/2 is able to translocate to the cell nucleus and then to promote the growth of the PDAC cell line^29,64–66^. NTS-mediated PDAC cell growth and proliferation are pharmacologically inhibited by SR48692, a NTS receptor antagonist^60^. A NTSR-specific Positron Emission Tomography (PET) has been developed thanks to a NTSR-directed fluorinated ligand. This method has demonstrated its diagnostic capacity in evaluating NTRS1-positive PDAC. Furthermore, a personalized NTRS-targeted therapy using PET has been created^67,68^ which has shown its feasibility and whose preliminary results are encouraging^69^.

Sortilin has been detected in PDAC cells in which NTS triggers pro-tumor and pro-metastatic effects^70^. NTS modulates the migration properties of human PDAC cells in vitro. These effects are mediated through NTS binding to sortilin and depend of whether the cells migrate individually or collectively. Indeed, migration levels of collectively migrating PDAC are decreased whereas those of individually migrating cells are increased. Mechanistically, expression of integrins is altered, resulting in modifications of both adhesion cell properties and actin cytoskeleton organization, mediated by NTS-dependent RhoGTPases activation^71^.

### Hepatic cancer

Hepatocellular carcinoma (HCC) is the third leading cause of cancer-related death worldwide with over 500,000 diagnosed patients each year^72^. Rapid progressive clinical course, poor response to conventional treatments and poor clinical outcomes are characteristic of HCC. Treatments available are surgery, percutaneous destruction or hepatic transplantation. Metastatic stages can be addressed with tyrosine kinase inhibitors (TKI; sorafenib, lenvatinib and regorafenib)^73^ or with transcatheter arterial chemoembolization. Most patients are diagnosed with advanced stages of the disease and, unfortunately, are not eligible for curative surgery^74^.

NTS and NTSR1 are coexpressed at high levels in more than 50% of HCC (NTSR1 being only weakly expressed in normal tissues); this coexpression correlates with aggressive biological behaviors: increase of EMT (epithelio-mesenchymal transition) features, e.g., decreased E-Cadherin expression and increased N-Cadherin and nuclear β-Catenin expressions. NTS and NTSR1 are thus associated with poor prognosis and clinical outcomes in HCC patients^75,76^. NTS effects on EMT induction and tumor invasion are mediated by aberrant activation of both the NTSR1 and Wnt/β-Catenin pathways. In vitro, EMT is abolished when NTSR1-overexpressing HCC cells are treated with the NTSR1 antagonist SR48692 or specific inhibitors of the Wnt/β-Catenin pathway as TSW119 or Dick kopf-1. In vivo, the same inhibitors prevent the formation of metastases in mice bearing NTSR1-overexpressing HCC xenografts^75^. NTSR1 activation by NTS, either produced in HCC cells expressing NTSR1 and NTS, or secreted—and transported - by the hepatic portal vein, leads to the stimulation of the Wnt/β-Catenin signaling pathway. Nuclear β-Catenin then activates the transcription of target genes, including *NTSR1* and *EGFR*. It results (i) a sustained autocrine signaling loop via NTSR1 and (ii) a sustained expression and activation of EGFR and its downstream effectors (such as ERK1/2), thus promoting tumor progression and invasion. On the other hand, the sustained expression and activation of EGFR presents somewhat beneficial effects since it restores and increases the cellular sensitivity to TKI such as erlotinib or sorafenib^76^.

Finally, the NTS pathway also plays key roles in the modulation of tumor microenvironment and the maintenance of HCC stem cells, through the regulation of pro-inflammatory/angiogenesis cytokines production and expression. In CD133-positive HCC stem cells, NTS stimulates and increases the expression and production of interleukin (IL)-8 and chemokine (C-X-C motif) ligand 1 (CXCL1), through activation of the RAF-1/ERK1/2 pathways, resulting in the building of the autocrine IL-8 signaling loop which promotes tumor angiogenesis, tumorigenesis and the self-renewal abilities of HCC cells^77^. Mechanistically, NTS stimulates IL-8 production and expression through activation of the MAPK and NFκB pathways. HCC-derived IL-8 induced upon NTS stimulation (i) attracts pro-inflammatory cells (CD28 + macrophages, CD66b + polynuclear neutrophils) to the local microenvironment, triggers an increased formation of pro-inflammatory cytokines and promotes phagocytosis, and (ii) stimulates the polarization of tumor-associated macrophages (TAMs), indirectly promoting EMT and enhances the invasive potential of HCC cells. All of these effects, mediated via activation of the NTS/IL-8 pathway, have been observed in vitro and in vivo; they are significantly reduced after the blockade of IL-8 and NTS receptors^78,79^.

In summary, NTS pathway plays crucial functions in the progression of HCC, by promoting invasion, EMT, metastasis formation, stem cells maintenance and by influencing the pro-oncogenic and inflammatory tumor microenvironment. In the future, NTSR1 could constitute a theranostic biomarker, whose evaluation might be useful to select TKI-eligible patients^76^. In addition, targeting the NTS/IL-8 pathway could also be promising in order to prevent HCC tumor progression, metastasis formation along with HCC stem cells maintenance.

### Colorectal cancer

With over 1.8 million new cases in 2018, CRC is the third most commonly occurring cancer worldwide (third in men and second in women) and the second cause of cancer-related death (over 880,000 deaths)^72^. Histological analyses have shown that Liberkhünien Adenocarcinomas are the most frequently found among CRC patients. Three distinct classifications of CRC have been established that take into account tumor heterogeneity; the different classes have been linked to patients’ prognosis^80^: (i) classification according to stages, i.e., the tumor damage of the different layers of the colonic wall (pTNM), (ii) histological classification, i.e., according to the state of differentiation of tumors, and, more recently, (iii) the molecular classification (cancer subtyping consortium or CMS). pTNM and histological classification are currently used, whereas CMS classification is rather oriented toward prognosis and personalized therapeutic decisions^81–83^. Therapeutic management of CRC is based on surgical resection when the tumor is well localized, accompanied with adjuvant chemotherapy (5-FU, oxaliplatin and folinic acid) for high-risk stage II and stage III patients, that is to say patients whose risk factors are associated with a high recurrence rate and a poor prognosis [lymphovascular invasion, *BRAF*^*V600E*^ hotspot mutation, MSS status^84^, KRAS mutated, presence of signet ring cell or mucinous histologic subtypes^85^]. For patients with advanced and metastatic CRC, chemotherapy “called neoadjuvant” is associated with treatments that mainly target the vascular endothelial growth factor and the epidermal growth factor (EGF) pathways^86^. Despite improvements in screening campaigns, earlier patient diagnosis, better prognosis evaluation and therapeutic management, 4 out of 5 CRC patients are still diagnosed at too advanced a stage, so they are unlikely to benefit from surgery. In 70% of cases, diagnosis is made at stages II or III and one-third of the treated patients suffer from recurrence within 2 years, which irreparably leads to the formation of metastases^87–89^. Therefore, new biomarkers are needed to allow (i) earlier detection of CRC and (ii) better, more specific and more efficient treatments, in order to overcome CRC and current therapeutic resistances.

Concerning diagnosis, a clinical study found that plasmatic NTS rate higher than 54.47 pg/mL, with a sensitivity of 77% and a specificity of 90%, could differentiate patients with CRC from controls^90^. More prospective cohorts are necessary to confirm this result.

Focusing on *NTSR1*, its promoter methylation^91^ or acetylation^41^ status could explain high NTSR1 gene and protein expression level in CRC. In a nutshell, the characterization of both NTSR1 gene and protein expressions could confirm the diagnosis of CRC and link it to a specific sub-type, thus forming a molecular signature. It is noteworthy that it is deacetylation of *NTSR1* promoter by histone deacetylase inhibitors (HDACi, e.g., sodium butyrate), (i) that alters NTSR1 mRNA and protein expression as well as its promoter activity. This is mediated through GSK3-β nuclear expression increase, (ii) with a decrease in ERK1/2 activation induced by NTS and (iii) consequently leads to the attenuation of the expression of some NTS-induced genes involved in colon tumorigenesis, cell proliferation and malignant transformation (e.g., *COX2*, *c-myc*, and *IL-8*). These results highlight that downregulation of *NTSR1* may represent a promising mechanism to target CRC through either HDACi or GSK3-β and ERK1/2 inhibitors^41^.

NTS pathway involvement in CRC tumor growth and cell proliferation has been highlighted since more than 20 years ago^23,28,40,92–94^. The majority of CRC cells are sensitive to NTS, which triggers growth responses by preferentially interacting with NTSR1 and/or sortilin, these two receptors being expressed in all CRC cells^70^. In vitro study published in 2015, focusing on human colorectal cancer cell lines corresponding to different colorectal cancer stages, showed variable NTS and NTSR1 expressions, no expression of NTSR2 and constant NTSR3 expression^95^. NTSR1 expression in adenocarcinoma is correlated with diffuse cytoplasmic or nuclear β-catenin localization^40^. Early stages of CRC development are characterized by mutations in the *APC* gene leading to a reinforced nuclear localization and increased expression of TCF/β-catenin. TCF/β-catenin then binds to the *NTSR1* promoter region and consequently triggers expression of this gene^40^. Reciprocally, NTSR1 also regulates β-Catenin activation and function^40,96,97^. NTS, through its binding to NTSR1, is able to stimulate the MAPK-ERK1/2 downstream signaling pathway which promotes the expression and activation of transcription factors such as *c-fos*, a member of AP-1 complex, and *Elk1*, which activates genes that are involved in cell growth and proliferation. NTSR1 inhibition by a selective antagonist (SR48692) inhibits tumor growth in vivo and cell proliferation in vitro, thus confirming the oncogenic effects of NTS which acts in both the autocrine and paracrine ways^98^. Moreover, signaling mechanisms mediating NTS effects involve multiple pathways and are cellular-dependent. In CRC (and contrary to PDAC), NTS can stimulate the ERK1/2 and AKT pathways through two different ways depending on cell lines^99^. In HT29, NTS-induced ERK1/2 and AKT activation is dependent on EGFR transactivation, but at the same time is PKC-independent. EGFR inhibition by gefitinib blocks NTS-induced activation of both the ERK1/2 and AKT pathways^64^. In HCT116, ERK1/2 activation and subsequent DNA synthesis are PKC-dependent, while PI3K/AKT activation is mainly EGFR transactivation-dependent^64^. Rho GTPases family which includes RhoA, Rac1 and Cdc42, are also key proteins involved in NTS oncogenic effects. NTS induces the expression of IL-8, in nontransformed human colonic epithelial cells and in CRC cells, and expression and secretion of IL-8 are both mediated through RhoGTPases-dependent NFκB activation. While these mechanisms are complex and not yet well understood, it has been observed that: (i) NTS rapidly activates the RhoGTPases RhoA, Rac1 and Cdc42, (ii) these RhoGTPases activated by NTS are necessary to activate the transcription factor NFκB and (iii) thus NFκB activated is required to trigger IL-8 production, expression and secretion^100^. In addition to RhoGTPases, IL-8 secretion would also depend on the release of intracellular Ca^2+^ and RAS-MEK1/2-ERK1/2 activation, the both of them being induced upon NTS stimulation. Indeed, the *IL-8* promoter region contains binding sites for NFκB, but also for c-jun and c-fos (AP-1 complex) which are both activated downstream of ERK1/2 activation^101^. While the NTS pathway could possibly play a role in the modulation of CRC microenvironment through IL-8 regulation^102,103^, as compared with HCC, this hypothesis deserves further investigation.

**Fig. 3:**
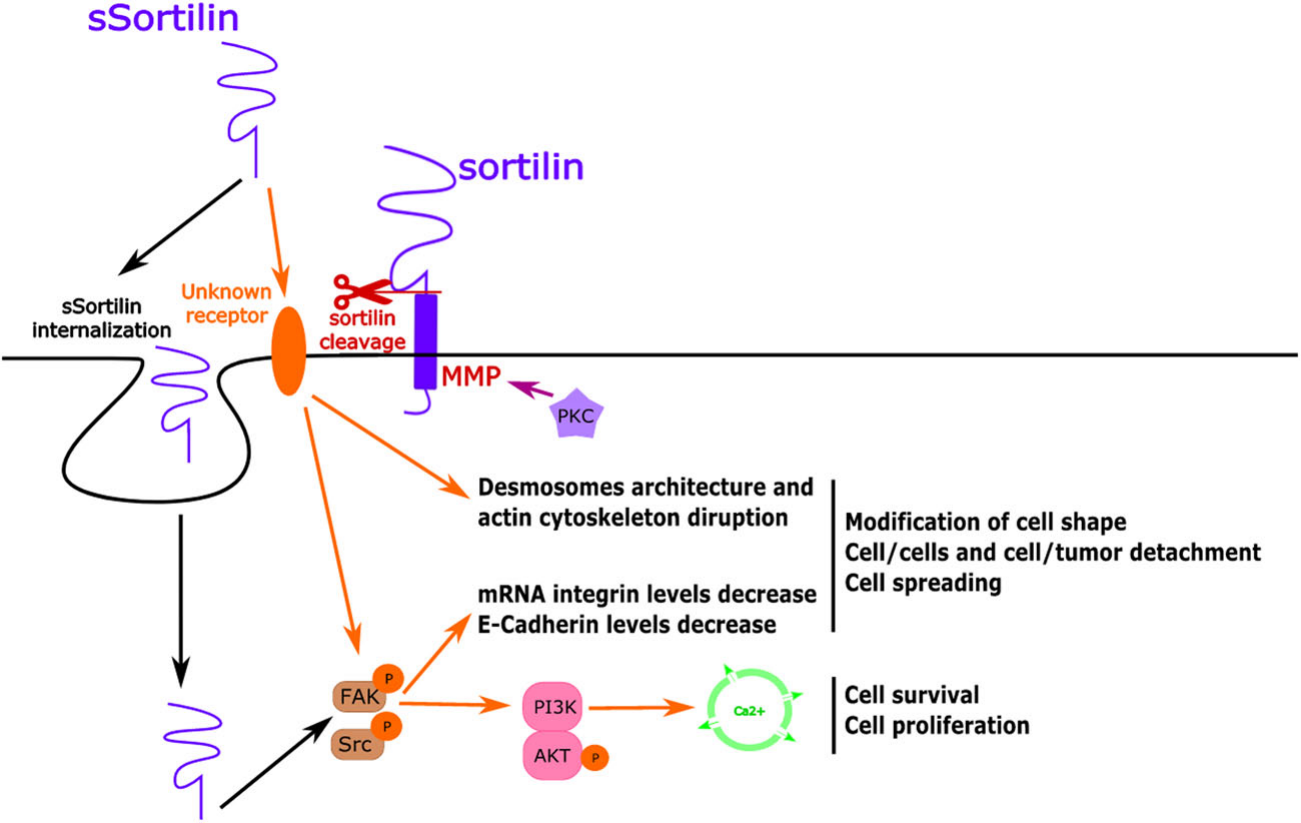
Sortilin and its soluble form (sSortilin) in colorectal cancer.

Expressed by the majority of CRC cells, the heterodimer NTSR1/sortilin modulates intracellular responses induced by NTS^34,104^. In the absence of NTS, this heterodimer is largely expressed at the plasma membrane. In the presence of NTS, it is internalized, leading to a decrease of membrane-bound NTSR1 while the total amount of membrane-bound sortilin remains unchanged. Moreover, NTSR1 remains bound to sortilin during internalization. Mechanistically, heterodimerization changes the functional properties of NTSR1 but not its ability to bind NTS. Indeed, ERK1/2 activation induced by NTS is lower in cells coexpressing both NTSR1 and sortilin. These changes in signaling responses induced by NTS between cells coexpressing NTSR1/sortilin and those expressing NTSR1 alone, would result from the conformational changes of NTSR1 induced by its binding to sortilin. Sortilin binding would hinder further association of NTSR1 with intracellular proteins (β-arestin and G protein-q), thus perturbing activation of downstream signaling pathways^34^. However, NTS/NTSR1/sortilin complex internalization would be necessary for ERK1/2 activation but not for IP3 formation and PKC activation, ERK1/2 and PKC pathways being activated upon NTS stimulation from independent mechanisms^104^. Finally, sortilin embedded in the plasma membrane can be cleaved (by shedding of its extracellular domain) via PKC-dependent MMP activation upon external stimuli, resulting in the formation of sSortilin, a functional soluble form of the protein. sSortilin is then either internalized or released in the extracellular medium. In this later case, in an autocrine and paracrine manner, sSortilin stimulates a yet unknown receptor and promotes the modification of cancer cell shape, followed by initiation of their separation and spreading^14,15^ (Fig. 3):

- sSortilin/unknown receptor complex promotes a disruption of desmosome architecture and a disorganization of actin filaments.
- sSortilin/unknown receptor complex stimulates FAK/Src pathways to increase integrin mRNA levels and to decrease E-Cadherin protein expression, leading to the detachment of the cell from other cells and from the extracellular matrix, thus leading to the formation of metastasis.
- sSortilin/unknown receptor complex activates FAK/Src pathways which can also stimulate PI3K/AKT activation and Ca^2+^ release, promoting cell survival and cell proliferation.

Altogether these data suggest that NTSR1 overexpression may represent an early event of colonic carcinogenesis, promoting CRC development and progression^97^, and might be a promising diagnostic and prognostic biomarker, and a potential therapeutic target to overcome chemotherapeutic resistances^105^. In addition, sSortilin displays key roles in the first step of the metastatic process^15^. Therefore, a better understanding of the molecular mechanisms involved in its functions could allow its targeting to prevent metastasis formation in CRC. In this way, the blockade of sortilin cleavage through targeting of MMP activity constitutes a new challenge: in vitro, the metalloprotease ADAM10 inhibitor (BB3103) totally blocked sSortilin production in the HT29 cell line^13^. Thus, targeting MMP to prevent both sSortilin and metastasis formation is promising^106^, but requires more investigations such as the development of selective inhibitors for specific MMPs and for each subtype of colorectal cancer.

## Discussion and remarks

NTS pathway displays important anti-apoptotic and pro-oncogenic effects, associated with tumor development, progression and metastasis formation in many types of cancer, e.g., digestive cancers. However, nothing has yet been found about NTS involvement in oesophageal cancer. Expression of the couple NTS/NSTR appears to promote tumor progression and aggressiveness in digestive cancers, and more especially colorectal cancer. More clinical studies are needed to correlate each subtype of CRC with the expression levels of both NTS and NTSR. Indeed, these levels could constitute signatures of each subtype of CRC, thus allowing to define the prognosis of each entity and to trigger personalized therapies in a way similar to what has been done for breast cancer in relation with the HER2 status^107^.

While NTSR2 expression has not been found in any of the digestive cancers, NTSR3/sortilin has mainly been studied and described in PDAC and CRC. This third NTSR plays key roles in the first step of the metastatic process, promoting EMT induction, cell migration and invasion. In CRC, sortilin, as well as its soluble form (sSortilin), may constitute a promising diagnostic, prognostic and therapeutic resistance biomarker. Its screening and targeting would thus be useful in clinical applications. However, more investigations are needed to better understand the molecular mechanisms involved in sortilin/sSortilin-mediated oncogenic effects in digestive cancers.

NTS and its high-affinity receptor NTSR1 are the most described and studied in digestive cancers. Both of them are overexpressed in tumors and this overexpression is associated with poor prognosis, since they are involved in each step of cancer progression. The numerous molecular mechanisms through which the NTS/NTSR1 complex mediates oncogenic effects are complex and different in each cancer cell-type and thus they must be more precisely and individually elucidated (Table 1). Nevertheless, NTS and NTSR1 might be very promising diagnostic, prognostic and therapeutic targets. Their screening and targeting may be useful for clinical applications by acting on primary tumors and migrating cancer cells, but also on the tumor microenvironment via the modulation of the IL-8 pathway, as observed in HCC and CRC. Indeed, production and secretion of IL-8 by tumor cells are induced by NTS and have an impact upon the tumor microenvironment. Besides the pro-inflammatory effects, IL-8 also triggers EMT and promotes cancer cell migration and invasion, as well as HCC stem cells maintenance. In addition, IL-8 attracts some stromal cells from the tumor microenvironment, e.g., neutrophils and TAMs, which also contribute to tumor progression by inducing EMT. Therefore, targeted therapies blocking either NTSR1 or IL-8 receptors may be effective to inhibit EMT process, thus preventing tumor progression and metastasis. In view of the importance of tumor microenvironment actors in cancer progression and of the growing emergence of immunotherapy, it becomes of the greatest interest to understand how NTS could influence the tumor microenvironment of digestive cancers and more particularly the perineural invasion (the network of peripheral nerves which connects the TME to the central nervous system, also involved in cancer cell dissemination)^108^. NTSR1 blocking becomes a major challenge in many studies. Several options have been described and tested in vitro and/or in vivo in digestive cancer models. Among them, a NTSR1 pharmacological antagonist (SR48692) has proved interesting, by inhibiting tumor growth and cancer cell proliferation and survival. Moreover, HDACi, e.g., NaBT, suppresses endogenous NTSR1 expression with efficient antitumor effects in CRC cell lines. Interestingly, studies have also demonstrated that curcumin, a naturally occurring polyphenolic pigment with chemopreventive and/or chemotherapeutic properties, inhibits the NTS/IL-8 pathway and thus CRC HCT116 cell line migration^103^.

**Table 1:** NTS, NTSR, downstream signaling pathways and main molecular mechanisms related to primary tumors, EMT and tumor microenvironement, and involved in each of the four digestive cancers described (GC, PDAC, HCC, and CRC).

	NTS and receptors	Activated signaling pathways, downstream stimulated effectors and upregulated genes	Tumor microenvironment modulation (NTS/IL-8)
GC	NTS (secreted), NTSR1	- PKC - ERK1/2 - PI3K/AKT	Not described
PDAC	1- NTS, NTSR1 2- NTS/sortilin	- PKC-dependent ERK1/2 - PI3K/AKT RhoGTPases	Not described
HCC	NTS, NTSR1	Wnt/β-catenin → *NTSR1, EGFR*	Stem cells maintenance RAF-1, ERK1/2 → *IL-8, CXCL1* EMT, cancer, and stromal cell infiltration within tumors MAPK, NFκB → *IL-8*
CRC	1- NTS, NTSR1 2- sortilin/sSortilin (secreted)	- PKB, β-catenin → *Cyclin D1* - Ca^2+^ release - RhoGTPases, NFκB → *IL-8* - AKT by EGFR-transactivation dependent (HT29, HCT116) - MAPK-ERK1/2 by EGFR-transactivation dependent (HT29) or PKC-activation dependent (HCT116) → AP-1, Elk1, Egr1 activation → *IL-8, EGFR* - FAK/Src - Ca^2+^ release	Not described

*CRC* colorectal cancer, *EGFR* epidermal growth factor receptor, *Egr-1* early growth response protein 1, *ERK1/2* extracellular signal–regulated kinases 1/2, *FAK* focal adhesion kinase, *GC* gastric cancer, *HCC* hepatocellular carcinoma, *IL* interleukin, *MAPK* mitogen-activated protein kinase, *NFκB* nuclear factor-kappa B, *NTS* neurotensin, *NTSR* neurotensin receptor, *PDAC* pancreatic ductal adenocarcinoma, *PI3K* phosphatidylinositol 3-kinase, *PKB* protein kinase B, *PKC* protein kinase C, *UR* unknown receptor.

To summarize, this review focused on oncogenic roles of NTS and its receptors in digestive cancers and highlighted their use as putative diagnostic, prognostic and therapeutic biomarkers. NTS/NTSR1 is the best described and studied couple of molecules: the both of them are overexpressed in digestive cancers and are associated with poor prognosis. Thus, NTS and NTSR1 are promising candidates for digestive cancers clinical screening and targeting. Recently, some studies conducted on PDAC^67,69,109–111^ and CRC^112,113^ have developed radiopharmaceuticals targeting NTSR1 for diagnostic and therapeutic application. Radiolabeled peptide and non-peptide tracers have been designed which are promising PET imaging probes for diagnosis, allowing the discrimination between patients with PDAC and those who only suffer from pancreas inflammation. In addition, a radiopharmaceutical treatment with NTSR1 antagonist has recently shown its feasibility and has led to encouraging results from a symptomatic and oncological point of view, albeit with a small cohort size (13 patients)^69^. The clinical trial NCT03525392, is presently recruiting patients with solid tumors (in particular, gastric cancers, CRC and PDAC) in order to evaluate the safety and efficacy of a radiopharmaceutical treatment using the radiolabeled NTSR1antagonist 177Lu-3BP-227.

These recent findings must be developed in the future and they will undoubtedly open new promising insights in NTS pathway targeting in digestive cancers.

## References

[1] Carraway R, Leeman SE (1973). The isolation of a new hypotensive peptide, neurotensin, from bovine hypothalami. J. Biol. Chem..

[2] Carraway R, Leeman SE (1976). Characterization of radioimmunoassayable neurotensin in the rat. Its differential distribution in the central nervous system, small intestine, and stomach. J. Biol. Chem..

[3] Ferris CF, Carraway RE, Hammer RA, Leeman SE (1985). Release and degradation of neurotensin during perfusion of rat small intestine with lipid. Regul. Pept..

[4] Mustain WC, Rychahou PG, Evers BM (2011). The role of neurotensin in physiologic and pathologic processes. Curr. Opin. Endocrinol. Diabetes Obes..

[5] Zhao D, Pothoulakis C (2006). Effects of NT on gastrointestinal motility and secretion, and role in intestinal inflammation. Peptides.

[6] Kitabgi P (1977). Neurotensin: specific binding to synaptic membranes from rat brain. Proc. Natl Acad. Sci..

[7] Morris NJ (1998). Sortilin is the major 110-kDa protein in GLUT4 vesicles from adipocytes. J. Biol. Chem..

[8] Mazella J (1998). The 100-kDa neurotensin receptor Is gp95/Sortilin, a non-g-protein-coupled receptor. J. Biol. Chem..

[9] Petersen CM (1997). Molecular identification of a novel candidate sorting receptor purified from human brain by receptor-associated protein affinity chromatography. J. Biol. Chem..

[10] Evers BM (1992). Neurotensin stimulates growth of colonic mucosa in young and aged rats. Gastroenterology.

[11] Glerup S, Nykjaer A, Vaegter CB (2014). Sortilins in neurotrophic factor signaling. Handb. Exp. Pharmacol..

[12] Talbot H (2019). Regulatory roles of sortilin and SorLA in immune-related processes. Front. Pharmacol..

[13] Navarro V, Vincent J-P, Mazella J (2002). Shedding of the luminal domain of the neurotensin receptor-3/sortilin in the HT29 cell line. Biochem. Biophys. Res. Commun..

[14] Massa F (2013). Focal adhesion kinase dependent activation of the PI3 kinase pathway by the functional soluble form of neurotensin receptor-3 in HT29 cells. Int. J. Biochem. Cell Biol..

[15] Béraud-Dufour S (2016). Focal adhesion kinase-dependent role of the soluble form of neurotensin receptor-3/sortilin in colorectal cancer cell dissociation. Int. J. Mol. Sci..

[16] Crcss AS, Azzopardi JG, Krausz T, Noorden SV, Polak JM (1985). A morphological and immunocytochemical study of a distinctive variant of ductal carcinoma in-situ of the breast. Histopathology.

[17] Dupouy S (2009). The Neurotensin Receptor-1 Pathway Contributes to Human Ductal Breast Cancer Progression. PLoS ONE.

[18] Ouyang Q (2017). Oncogenic role of neurotensin and neurotensin receptors in various cancers. Clin. Exp. Pharmacol. Physiol..

[19] Qiu S (2019). Exploratory analysis of plasma neurotensin as a novel biomarker for early detection of colorectal polyp and cancer. Horm. Cancer.

[20] Ye, Y. et al. Neurotensin, a Novel Messenger to Cross-Link Inflammation and Tumor Invasion via Epithelial-Mesenchymal Transition Pathway. Int. Rev. Immunol.1–11 (2014). 10.3109/08830185.2014.952412. 10.3109/08830185.2014.952412 25215420

[21] Wu Z, Martinez-Fong D, Trédaniel J, Forgez P (2013). Neurotensin and its high affinity receptor 1 as a potential pharmacological target in cancer therapy. Front. Endocrinol..

[22] Dorsam RT, Gutkind JS (2007). G-protein-coupled receptors and cancer. Nat. Rev. Cancer.

[23] Liu, Aihua, Zuo, Z., Liu, Linlin & Liu, Lihua. Down-regulation of NTSR3 inhibits growth, metastasis, and PI3K/AKT and MAPK signaling pathways in colorectal cancer cells. *Biochem. Cell Biol*. (2020). 10.1139/bcb-2019-0351. 10.1139/bcb-2019-0351 32125883

[24] Forgez, P. Anti-neurotensin long fragment antibodies and uses thereof. United States patent 10, 174, 106B2 (2019).

[25] Sakamoto T (1984). Role of neurotensin in pancreatic secretion. Surgery.

[26] Vincent J-P, Mazella J, Kitabgi P (1999). Neurotensin and neurotensin receptors. Trends Pharmacol. Sci..

[27] Zhao D (2007). Neurotensin stimulates expression of early growth response gene-1 and EGF receptor through MAP kinase activation in human colonic epithelial cells. Int. J. Cancer J. Int. Cancer.

[28] Ehlers RA, Bonnor RM, Wang X, Hellmich MR, Evers BM (1998). Signal transduction mechanisms in neurotensin-mediated cellular regulation. Surgery.

[29] Ehlers RA, Zhang Y, Hellmich MR, Evers BM (2000). Neurotensin-mediated activation of MAPK pathways and AP-1 binding in the human pancreatic cancer cell line, MIA PaCa-2. Biochem. Biophys. Res. Commun..

[30] Mazella J, Vincent J-P (2006). Functional roles of the NTS2 and NTS3 receptors. Peptides.

[31] Martin S, Vincent J-P, Mazella J (2003). Involvement of the neurotensin receptor-3 in the neurotensin-induced migration of human microglia. J. Neurosci..

[32] Nielsen MS, Jacobsen C, Olivecrona G, Gliemann J, Petersen CM (1999). Sortilin/neurotensin receptor-3 binds and mediates degradation of lipoprotein lipase. J. Biol. Chem..

[33] Wilson CM (2014). The implications of Sortilin/Vps10p domain receptors in neurological and human diseases. CNS Neurol. Disord. - Drug Targets..

[34] Martin S, Navarro V, Vincent JP, Mazella J (2002). Neurotensin receptor-1 and -3 complex modulates the cellular signaling of neurotensin in the HT29 cell line. Gastroenterology.

[35] Wilson CM (2014). Sortilin mediates the release and transfer of exosomes in concert with two tyrosine kinase receptors. J. Cell Sci..

[36] Blondy S (2019). Neurotrophins and their involvement in digestive cancers. Cell Death Dis..

[37] Glerup, S., Nykjaer, A. & Vaegter, C. B. Sortilins in Neurotrophic Factor Signaling. In *Neurotrophic Factors* (eds. Lewin, G. R. & Carter, B. D.) vol. 220 165–189 (Springer Berlin Heidelberg, 2014). 10.1007/978-3-642-45106-5_7 24668473

[38] Meldolesi J (2017). Neurotrophin receptors in the pathogenesis, diagnosis and therapy of neurodegenerative diseases. Pharmacol. Res..

[39] Dupouy S (2011). The potential use of the neurotensin high affinity receptor 1 as a biomarker for cancer progression and as a component of personalized medicine in selective cancers. Biochimie.

[40] Souazé F (2006). Neurotensin receptor 1 gene activation by the Tcf/β-catenin pathway is an early event in human colonic adenomas. Carcinogenesis.

[41] Wang X, Jackson LN, Johnson SM, Wang Q, Evers BM (2010). Suppression of neurotensin receptor type 1 expression and function by histone deacetylase inhibitors in human colorectal cancers. Mol. Cancer Ther..

[42] Abbaci, A. et al. Neurotensin receptor type 2 protects B-cell chronic lymphocytic leukemia cells from apoptosis. *Oncogene* (2017). 10.1038/onc.2017.365. 10.1038/onc.2017.365 PMC5808079 29059151

[43] Saada S (2012). Differential expression of neurotensin and specific receptors, NTSR1 and NTSR2, in normal and malignant human B lymphocytes. J. Immunol..

[44] Ayala-Sarmiento AE, Martinez-Fong D, Segovia J (2015). The internalization of neurotensin by the low-affinity neurotensin receptors (NTSR2 and vNTSR2) activates ERK 1/2 in glioma cells and allows neurotensin-polyplex transfection of tGAS1. Cell. Mol. Neurobiol..

[45] Gao A (2017). Implications of sortilin in lipid metabolism and lipid disorder diseases. DNA Cell Biol..

[46] Goettsch C, Kjolby M, Aikawa E (2018). Sortilin and its multiple roles in cardiovascular and metabolic diseases. Arterioscler. Thromb. Vasc. Biol..

[47] Carlo A-S (2013). The pro-neurotrophin receptor sortilin is a major neuronal APOE receptor for catabolism of amyloid-β peptide in the brain. J. Neurosci..

[48] Wilson CM (2016). A new role under sortilin’s belt in cancer. Commun. Integr. Biol..

[49] Sitarz R (2018). Gastric cancer: epidemiology, prevention, classification, and treatment. Cancer Manag. Res..

[50] Stomach Cancer Surgery | Stomach Cancer Operation. https://www.cancer.org/cancer/stomach-cancer/treating/types-of-surgery.html.

[51] Bang Y-J (2010). Trastuzumab in combination with chemotherapy versus chemotherapy alone for treatment of HER2-positive advanced gastric or gastro-oesophageal junction cancer (ToGA): a phase 3, open-label, randomised controlled trial. Lancet.

[52] Abrahao-Machado LF, Scapulatempo-Neto C (2016). HER2 testing in gastric cancer: An update. World J. Gastroenterol..

[53] Zhou Z (2015). The significance of NTR1 expression and its correlation with β-catenin and EGFR in gastric cancer. Diagn. Pathol..

[54] Akter H, Yoon JH, Yoo YS, Kang M-J (2018). Validation of neurotensin receptor 1 as a therapeutic target for gastric cancer. Mol. Cells.

[55] Zygulska AL, Furgala A, Kaszuba-Zwoińska J, Krzemieniecki K, Gil K (2019). Changes in plasma levels of cholecystokinin, neurotensin, VIP and PYY in gastric and colorectal cancer – Preliminary results. Peptides.

[56] Akter H (2015). Activation of matrix metalloproteinase-9 (MMP-9) by neurotensin promotes cell invasion and migration through ERK pathway in gastric cancer. Tumor Biol..

[57] Drouillard A, Manfredi S, Lepage C, Bouvier A-M (2018). Épidémiologie du cancer du pancréas. Bull. Cancer.

[58] Rahib L (2014). Projecting cancer incidence and deaths to 2030: the unexpected burden of thyroid, liver, and pancreas cancers in the United States. Cancer Res..

[59] Reubi J, Waser B, Friess H, Buchler M, Laissue J (1998). Neurotensin receptors: a new marker for human ductal pancreatic adenocarcinoma. Gut.

[60] Wang J-G, Li N-N, Li H-N, Cui L, Wang P (2011). Pancreatic cancer bears overexpression of neurotensin and neurotensin receptor subtype-1 and SR 48692 counteracts neurotensin induced cell proliferation in human pancreatic ductal carcinoma cell line PANC-1. Neuropeptides.

[61] Wang L (2000). Neurotensin receptor-1 mRNA analysis in normal pancreas and pancreatic disease. Clin. Cancer Res..

[62] Körner M, Waser B, Strobel O, Büchler M, Reubi JC (2015). Neurotensin receptors in pancreatic ductal carcinomas. EJNMMI Res..

[63] Guha S, Rey O, Rozengurt E (2002). Neurotensin induces protein kinase C-dependent protein kinase D activation and DNA synthesis in human pancreatic carcinoma cell line PANC-1. Cancer Res..

[64] Müller KM (2011). Role of protein kinase C and epidermal growth factor receptor signalling in growth stimulation by neurotensin in colon carcinoma cells. BMC Cancer.

[65] Guha S, Lunn JA, Santiskulvong C, Rozengurt E (2003). Neurotensin stimulates protein kinase C-dependent mitogenic signaling in human pancreatic carcinoma cell line PANC-1. Cancer Res..

[66] Ryder NM, Guha S, Hines OJ, Reber HA, Rozengurt E (2001). G protein–coupled receptor signaling in human ductal pancreatic cancer cells: neurotensin responsiveness and mitogenic stimulation†. J. Cell. Physiol..

[67] Wang M (2018). Development of [18F]AlF-NOTA-NT as PET agents of neurotensin receptor-1 positive pancreatic cancer. Mol. Pharm..

[68] Hodolic M, Ambrosini V, Fanti S (2020). Potential use of radiolabelled neurotensin in PET imaging and therapy of patients with pancreatic cancer. Nucl. Med. Commun..

[69] Baum RP (2018). 177Lu-3BP-227 for neurotensin receptor 1–targeted therapy of metastatic pancreatic adenocarcinoma: first clinical results. J. Nucl. Med..

[70] Dal Farra C (2001). Involvement of the neurotensin receptor subtype NTR3 in the growth effect of neurotensin on cancer cell lines. Int. J. Cancer.

[71] Mijatovic T (2007). Neurotensin is a versatile modulator of in vitro human pancreatic ductal adenocarcinoma cell (PDAC) migration. Cell. Oncol. J. Int. Soc. Cell. Oncol..

[72] Bray F (2018). Global cancer statistics 2018: GLOBOCAN estimates of incidence and mortality worldwide for 36 cancers in 185 countries. CA. Cancer J. Clin..

[73] Treatment of Liver Cancer, by Stage. https://www.cancer.org/cancer/liver-cancer/treating/by-stage.html.

[74] El-Serag HB (2011). Hepatocellular Carcinoma. N. Engl. J. Med..

[75] Ye Y (2016). NTS/NTR1 co-expression enhances epithelial-to-mesenchymal transition and promotes tumor metastasis by activating the Wnt/β-catenin signaling pathway in hepatocellular carcinoma. Oncotarget.

[76] Wu Z (2017). Neurotensin regulation induces overexpression and activation of EGFR in HCC and restores response to erlotinib and sorafenib. Cancer Lett..

[77] Tang KH (2012). CD133+ liver tumor-initiating cells promote tumor angiogenesis, growth, and self-renewal through neurotensin/interleukin-8/CXCL1 signaling. Hepatology.

[78] Xiao P (2018). Neurotensin/IL-8 pathway orchestrates local inflammatory response and tumor invasion by inducing M2 polarization of Tumor-Associated macrophages and epithelial-mesenchymal transition of hepatocellular carcinoma cells. Oncoimmunology.

[79] Yu J (2013). Dysfunctional Activation of Neurotensin/IL-8 Pathway in Hepatocellular Carcinoma Is Associated with Increased Inflammatory Response in Microenvironment, More Epithelial Mesenchymal Transition in Cancer and Worse Prognosis in Patients. PLoS ONE.

[80] Singh, M. P., Rai, S., Pandey, A., Singh, N. K. & Srivastava, S. Molecular subtypes of colorectal cancer: An emerging therapeutic opportunity for personalized medicine. *Genes Dis*. (2019). 10.1016/j.gendis.2019.10.013. 10.1016/j.gendis.2019.10.013 PMC8099693 33997160

[81] Barresi V, Reggiani Bonetti L, Ieni A, Caruso RA, Tuccari G (2015). Histological grading in colorectal cancer: new insights and perspectives. Histol. Histopathol..

[82] Guinney J (2015). The consensus molecular subtypes of colorectal cancer. Nat. Med..

[83] Brierley, J. D., Gospodarowicz, Ma. K. & Wittekind, C. *TNM Classification of Malignant Tumours*, 8th Edition. 83. (Wiley publisher, 2017).

[84] Seppälä TT (2015). Combination of microsatellite instability and BRAF mutation status for subtyping colorectal cancer. Br. J. Cancer.

[85] Liska D (2017). Incidence, patterns, and predictors of locoregional recurrence in colon cancer. Ann. Surg. Oncol..

[86] Roock WD, Vriendt VD, Normanno N, Ciardiello F, Tejpar S (2011). KRAS, BRAF, PIK3CA, and PTEN mutations: implications for targeted therapies in metastatic colorectal cancer. Lancet Oncol..

[87] Stein A, Atanackovic D, Bokemeyer C (2011). Current standards and new trends in the primary treatment of colorectal cancer. Eur. J. Cancer.

[88] André T (2015). Adjuvant fluorouracil, leucovorin, and oxaliplatin in stage II to III colon cancer: updated 10-year survival and outcomes according to braf mutation and mismatch repair status of the MOSAIC Study. J. Clin. Oncol..

[89] André, T. & Cohen, R. Cancer colorectal: traitements adjuvants. *FMC-HGE.* https://www.fmcgastro.org/texte-postu/postu-2018-paris/cancer-colorectal-traitements-adjuvants/ (2018).

[90] Sgourakis G (2014). The combined use of serum neurotensin and IL-8 as screening markers for colorectal cancer. Tumour Biol..

[91] Kamimae S (2015). Epigenetic silencing of NTSR1 is associated with lateral and noninvasive growth of colorectal tumors. Oncotarget.

[92] Maoret J-J, Anini Y, Rouyer-Fessard C, Gully D, Laburthe M (1999). Neurotensin and a non-peptide neurotensin receptor antagonist control human colon cancer cell growth in cell culture and in cells xenografted into nude mice. Int. J. Cancer.

[93] Evers BM, Ishizuka J, Chung DH, Townsend CM, Thompson JC (1992). Neurotensin expression and release in human colon cancers. Ann. Surg..

[94] Qiu S (2017). A review of the role of neurotensin and its receptors in colorectal cancer. Gastroenterol. Res. Pract..

[95] Kim JT, Weiss HL, Evers BM (2017). Diverse expression patterns and tumorigenic role of neurotensin signaling components in colorectal cancer cells. Int. J. Oncol..

[96] Fodde R, Smits R, Clevers H (2001). *APC*, signal transduction and genetic instability in colorectal cancer. Nat. Rev. Cancer.

[97] Gui X, Guzman G, Dobner PR, Kadkol SS (2008). Increased neurotensin receptor-1 expression during progression of colonic adenocarcinoma. Peptides.

[98] Gully D (1993). Biochemical and pharmacological profile of a potent and selective nonpeptide antagonist of the neurotensin receptor. Proc. Natl Acad. Sci. USA..

[99] Massa F, Tormo A, Béraud-Dufour S, Coppola T, Mazella J (2011). Neurotensin-induced Erk1/2 phosphorylation and growth of human colonic cancer cells are independent from growth factors receptors activation. Biochem. Biophys. Res. Commun..

[100] Zhao D (2003). Neurotensin stimulates IL-8 expression in human colonic epithelial cells through Rho GTPase-mediated NF-κB pathways. Am. J. Physiol. -Cell Physiol..

[101] Zhao D (2001). Signal transduction pathways mediating neurotensin-stimulated interleukin-8 expression in human colonocytes. J. Biol. Chem..

[102] Ning Y (2011). Interleukin-8 is associated with proliferation, migration, angiogenesis and chemosensitivity in vitro and in vivo in colon cancer cell line models. Int. J. Cancer.

[103] Wang X, Wang Q, Ives KL, Evers BM (2006). Curcumin inhibits neurotensin-mediated interleukin-8 production and migration of HCT116 human colon cancer cells. Clin. Cancer Res..

[104] Navarro V, Martin S, Mazella J (2006). Internalization-dependent regulation of HT29 cell proliferation by neurotensin. Peptides.

[105] Bugni JM (2012). The neurotensin receptor-1 promotes tumor development in a sporadic but not an inflammation-associated mouse model of colon cancer. Int. J. Cancer.

[106] Cathcart J, Pulkoski-Gross A, Cao J (2015). Targeting matrix metalloproteinases in cancer: bringing new life to old ideas. Genes Dis..

[107] Harbeck N, Gnant M (2017). Breast cancer. Lancet Lond. Engl..

[108] Jobling P (2015). Nerve–cancer cell cross-talk: a novel promoter of tumor progression. Cancer Res..

[109] Prignon A (2019). Preclinical evaluation of 68Ga-DOTA-NT-20.3: a promising pet imaging probe to discriminate human pancreatic ductal adenocarcinoma from pancreatitis. Mol. Pharm..

[110] Emrarian I, Sadeghzadeh N, Abedi SM, Abediankenari S (2018). New neurotensin analogue radiolabeled by 99m-technetium as a potential agent for tumor identification. Chem. Biol. Drug Des..

[111] Yin X (2017). Evaluation of neurotensin receptor1 as a potential imaging target in pancreatic ductal adenocarcinoma. Amino Acids.

[112] Lang C, Maschauer S, Hübner H, Gmeiner P, Prante O (2013). Synthesis and evaluation of a 18f-labeled diarylpyrazole glycoconjugate for the imaging of NTS1-positive tumors. J. Med. Chem..

[113] Jia Y, Zhang W, Fan W, Brusnahan S, Garrison J (2016). Investigation of the biological impact of charge distribution on a NTR1-targeted peptide. Bioconjug. Chem..

